# Treatment of traumatic sternal fractures with titanium plate internal fixation: a retrospective study

**DOI:** 10.1186/s13019-017-0580-x

**Published:** 2017-04-04

**Authors:** Yonghong Zhao, Yi Yang, Zongli Gao, Weiming Wu, Weiwei He, Tiancheng Zhao

**Affiliations:** Department of Thoracic Surgery, Shanghai Sixth People’s Hospital, Shanghai, 200233 People’s Republic of China

**Keywords:** Chest trauma, Sternal fracture, Nonunion, Internal fixation

## Abstract

**Background:**

This study aim to evaluate surgical procedures for titanium plate internal fixation of sternal fractures with displacement or nonunion.

**Methods:**

From January 2010 to December 2014, 64 patients with sternal fractures were treated with titanium plate internal fixation in the thoracic surgery department of the Shanghai Sixth People’s Hospital. Pain severity scale scores were analyzed preoperatively and postoperatively. All the patients had a 2-month follow-up for treatment evaluation.

**Results:**

The mean hospital length of stay was 16.89 days. Forty-five patients underwent surgery for combined injuries. A statistically significant difference (*P* < 0.05) was found between preoperative and postoperative pain severity scores (7.74 ± 0.89 vs. 3.80 ± 0.79, respectively). At follow-up, healing of the nonunion or fracture was confirmed in all the cases.

**Conclusion:**

The rigid titanium plate application ensured a safe and easy management of traumatic sternal fractures and nonunion with a good prognosis as compared with other methods.

## Background

Sternal fracture accounts for about 3–8% of admissions for thoracic trauma [1, 2]. It is not uncommon and is often caused by direct, frontal, blunt trauma to the sternum. Most sternal fractures heal with conservative management [1, 3, 4], but a few cases with instability or obvious displacement can lead to severe disabling conditions, including severe chest pain, dyspnea, persistent cough, and chest wall paradoxical motion.

The treatment most often used for this condition is corset fixation and bed rest for months, or steel wire fixation. The treatment often fails because of loss of tensile strength or wire cutout effect [5, 6]. Many authors reported the beneficial effect of plate internal fixation for sternal infection or nonunion after sternotomy. Sternal plating appears to be an effective treatment option for wound dehiscence associated with sternal instability [7–11]. The steel wire sealing technique is suitable for longitudinal sternotomy, but most traumatic sternal fractures are transverse fractures or nonunions. In these cases, internal fixation with a titanium locking plate is a better choice [12, 13].

We evaluated the status of sternal fractures by using computed tomography (CT) and three-dimensional (3-D) reconstruction imaging. Patients with sternal displacement or nonunion underwent surgery with a titanium plate, with screws rigidly fixed to the plate. The purpose of this study was to evaluate the therapeutic effect of this internal fixation technique.

## Methods

### Patients

From January 2010 to December 2014, 64 patients with sternal fractures were treated with titanium plate internal fixation in our thoracic surgery department. The operative criterion was a disabling nonunion or obvious displaced fracture of the sternum. The exclusion criteria were inability to provide informed consent or health status that ruled out general anesthesia.

### Materials

**Fig. 1:**
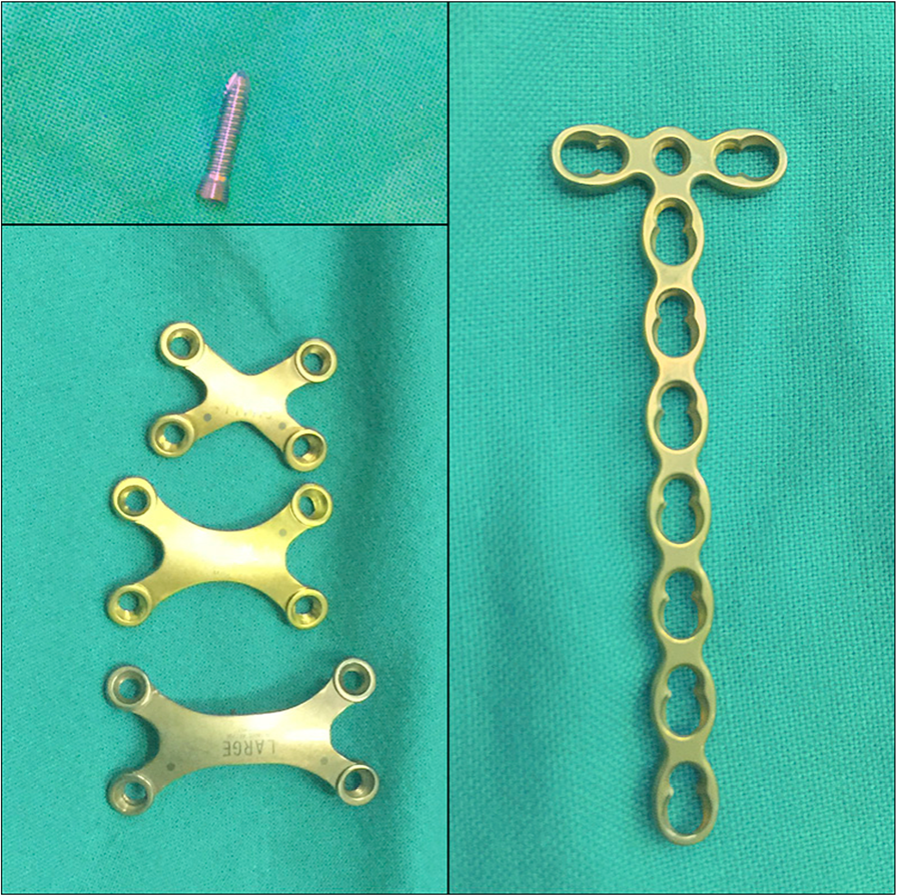
Different shapes of titanium plates

The internal fixation device used consisted of a titanium plate and screws. According to different fracture patterns, we could choose an X-shape, T-shape, or linear plate (Fig. 1). The overall thickness of the plate was 2.4 mm in all the patients. The implant was produced by DePuy Synthes, USA.

### Methods

**Fig. 2:**
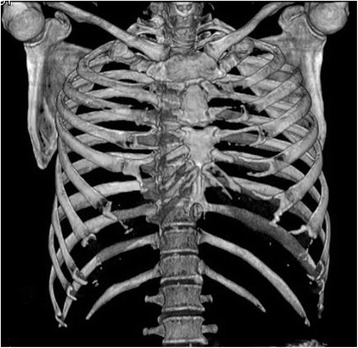
A sternal fracture with detached displacement and nonunion

All the patients underwent CT scan and 3-D reconstruction of the sternum preoperatively. This is useful in evaluating the fracture pattern and locating the fracture position. Figure 2 shows a sternal fracture with a detached displacement and nonunion 3 months after injury. Pain severity scale scores were measured both preoperatively and postoperatively. The patients were asked to grade their pain on a scale of 0 to 10, with 0 being “none” and 10 being the worst pain they had ever felt.

**Fig. 3:**
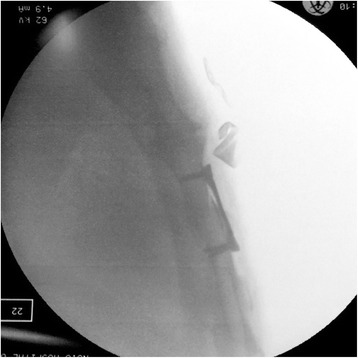
An intraoperative, portable wet-film radiography image of the sternum

**Fig. 4:**
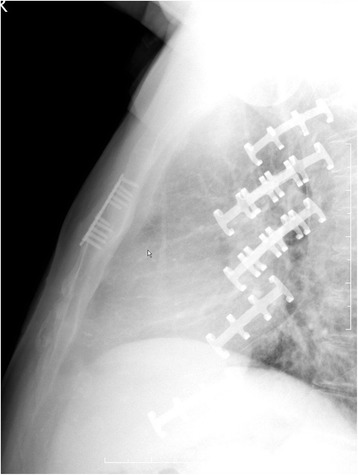
A patient 2 months after internal fixation for sternal and rib fractures

General anesthesia and single-lumen endotracheal intubation were suitable for the operation. The supine position and slight overextension of the chest wall were useful in repositioning the fracture. A longitudinal incision was made over the fracture, the fibrous tissue overlying the sternum was cleaned, and the fracture was then repositioned with a reduction clamp. In accordance with the fracture pattern, the appropriate plate was selected. Allogeneic bone fragments are used to fill in the nonunion gap. Portable wet film radiography of the sternum was performed intraoperatively to confirm proper plate placement and screw length (Fig. 3). The patients returned to their daily activities soon after surgery but were asked to restrict extreme movements of the chest. All the patients had a 2-month follow-up for evaluation of recovery by using radiography or CT. Figure 4 shows a patient 2 months after internal fixation for sternal and rib fractures.

The data were analyzed with SPSS version 19.0 (SPSS Inc., Chicago, IL, USA). The data with normal distribution were presented as mean ± standard deviation (SD) and independent sample *t* test, and enumeration data were expressed as a rate *χ* ^2^ test. *P* values of <0.05 were considered statistically significant.

## Results

**Fig. 5:**
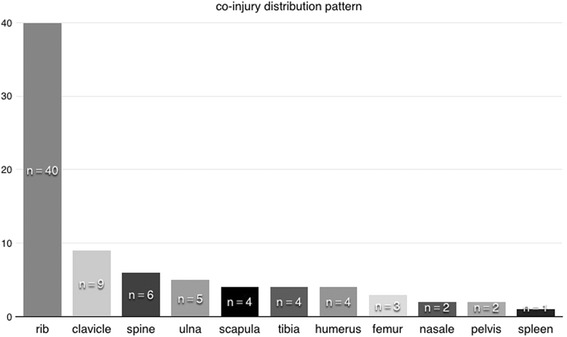
Co-injury distribution pattern

Sixty-four patients underwent the procedure, 51 of whom were male (80.1%). The mean age was 51 years (range: 21–71 years; Table 1). The mean duration between trauma and surgery was 7.25 days (range: 1–92 days). Of the patients, 50 (79.3%) had acute injuries and 13 (20.7%) had chronic nonunion after conservative therapy. Traffic accidents were the most common injury mechanism (55 patients, 87.3%). The remaining patients (8, 12.7%) were injured by impact with a heavy object. Forty-five patients had combined injures, detailed in Fig. 5. Rib fracture was the most common combined injury, concurrent with sternal fracture.

**Table 1:** General clinical characteristics of patients

	*n* = 64
Gender	
Male	51 (80.1%)
Female	13 (19.9%)
Age	51 (min 21, max 71)
Operation time (min)	42.62 ± 10.23
Hospital stay time (day)	16.89 ± 3.52

The mean operative time was 42.62 ± 10.23 min. No operation-related complications were observed. The mean hospital stay was 8.89 days, with 45 patients undergoing operation for combined injuries. A statistically significant difference (*P* < 0.05) was found between preoperative and postoperative pain severity scores (7.74 ± 0.89 vs. 3.80 ± 0.79, respectively). At follow-up, healing of the nonunion or fracture was confirmed in all the cases. No removal of metalwork was performed in the follow-up period. All the patients were followed up after operation for 6 months and underwent reevaluation for lung function with chest radiography, which revealed healed bones, callus formation, no nonunion, and displacement. No failure of internal fixation or migration of the internal fixation device was observed. All the patients were evaluated for pulmonary function before operation and on postoperative day 7 (FVC: forced vital capacity, FEV1: forced expiratory volume in 1 s). At the follow-up visit after 3 months, pulmonary function was evaluated again. The European Community of Coal and Steel (ECCS) formula was used to calculate the predictive value of the pulmonary function of the patients. We compared the measured and predicted values, and analyzed them. Studies have shown that the mean FVC values before operation and on postoperative day 7 accounted for 39.18% ± 9.18% and 52.91% ± 10.98% of the predicted values, respectively. FEV1 respectively accounted for 41.83% ± 14.87% and 63.11% ± 11.25% of the predicted values. Compared with pulmonary function before operation, that on postoperative day 7 significantly improved. The FVC and FEV1 at 3 months after operation accounted for 88.19% ± 8.82% and 85.26% ± 9.26% of the predicted values, respectively, and significantly improved from the preoperative values (*P* < 0.05; Table 2).

**Table 2:** Respiratory function and pain scale result

Clinical result of sternal internal fixation				
	Preoperation	Postoperation- day 7	Postoperation-3 month	*p*
FVC	39.18% ± 9.18%	52.91% ± 10.98%	88.19% ± 8.82%	<0.05
FEV1	41.83% ± 14.87%	63.11% ± 11.25%	85.26% ± 9.26%	<0.05
Pain Scale	7.74 ± 0.89	3.80 ± 0.79		<0.05

## Discussion

Stable sternal fractures without displacement or full cortical disruption can be managed conservatively, with treatments including corset fixation and bed rest. However, for sternal fractures with obvious displacement, conservative therapy frequently fails and leads to a sternal nonunion due to multidirectional motion, with tension and compression caused by respiration. Mayberry et al. [14] identified three key points for intervention as follows: (1) the presence of a sternal deformity, (2) the loss of sternal continuity for a period exceeding 6 weeks, and (3) the persistence of chest pain (between 2 and 8 weeks, in the opinion of most surgeons surveyed). Divisi et al. [13] stated that possible injury to the underlying vascular structures must also be considered. Steel wire fixation is widely used in longitudinal sternotomy but often fails in horizontal fractures and nonunion. Several methods have been investigated to overcome this problem. These included combining the wires with anchor plates to increase the tensile strength [1, 15, 16] and even intramedullary plating [17]. The number of cases using the above-described methods is still too small. These techniques seemed difficult because of the wide exposure of sternal fragments and the difficult positioning of the intramedullary pins.

In our clinical experience, titanium plate internal fixation is a fast, easy, and minimally invasive procedure. We noted the following advantages of this approach: (1) The exposure of the sternal fracture is minimal, without the need to expose the ribs and costal cartilage; thus, the risk of iatrogenic complications (intercostal and internal mammary artery injuries) is maximally reduced. (2) If the sternal face is not completely smooth, the titanium plate can easily be modeled according to the morphology of the fracture. Finally, (3) the locking screws offer a reliable and rigid fixation that can resist multidirectional tension. Precise depth measurement and intraoperative radiography can ensure that proper length screws are chosen. An X-shaped plate can be used for sternal fractures without nonunion. The linear plate prevents the movement of the sternal stumps, as it provides a rigid support for the sternum, with the screws anchored both above and below the fracture at the required distance. We do not recommend pulling the fracture components together into an assembly and using fixation to treat a sternal nonunion, as continuous and repeated shear force caused by respiration will loosen the fixation and lead to failure. We used allogeneic bone fragments to fill in the nonunion gap and then fixed the fracture with titanium plate, maintaining the sternum in a position of non-tension, with a satisfactory therapeutic result. Owing to the reduction of sternal fragment movement, chest pain was relieved rapidly and dramatically, as evidenced by the pain severity scores before and after surgery (7.74 ± 0.89 and 3.80 ± 0.79, respectively; *P* < 0.05). The patients had a shorter hospital mean length of stay of 8.89 days. The procedure resulted in satisfactory recovery, as 18 patients with isolated sternal fractures fully returned to their previous activity level by 3 months after operation.

## Conclusion

The use of a titanium plate and locking screws offers a reliable method for the successful treatment of sternal nonunion and dislocated fractures, and might also be considered for treatment of sternal dehiscence after median sternotomy. We believe that internal fixation is a proper surgical treatment technique for sternal fractures and nonunion.

## Abbreviations

3-D: 3-dimensional
CT: Computed tomography
ECCS: European Community of Coal and Steel
VC: Vital capacity
